# Interstitial ectopic pregnancy: A rare and difficult clinicosonographic diagnosis

**DOI:** 10.4103/0974-1208.44116

**Published:** 2008

**Authors:** R Rastogi, Meena GL, N Rastogi, V Rastogi

**Affiliations:** Yash Diagnostic Center, Yash Hospital and Research Center, Civil Lines, Kanth Road, Moradabad, Uttar Pradesh-244 001, India; 1 Govt. Medical College, Kota, Rajasthan, India

**Keywords:** Cornual, ectopic, interstitial, pregnancy

## Abstract

Ectopic pregnancy in the interstitial part of the fallopian tube is a rare event. This condition presents a challenge for clinical as well as radiological diagnosis. Although routine two-dimensional ultrasound can be suggestive, three-dimensional ultrasound is highly accurate in diagnosis. Hence, the authors report a rare case of interstitial ectopic pregnancy diagnosed preoperatively by three-dimensional ultrasound and managed laparoscopically.

## INTRODUCTION

Interstitial ectopic pregnancy is defined as the ectopic gestation developing in the uterine part of the fallopian tube. It is a rare event constituting only 5% of all tubal ectopic pregnancies and is associated with a high rate of complications.[1] The condition is difficult to diagnose, both clinically and sonographically. The authors therefore, present a rare case of interstitial ectopic pregnancy that was diagnosed by using 3D ultrasound and managed by laparoscopy.

## CASE REPORT

**Figure 1:**
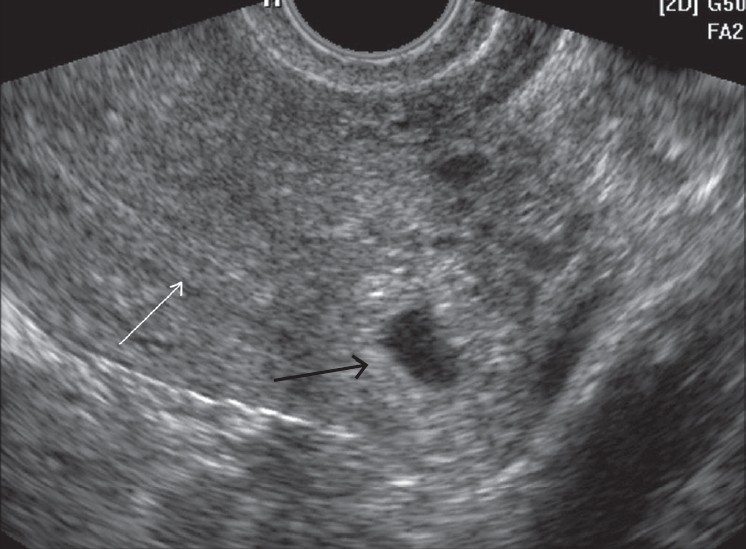
Axial 2D US scan at the level of the fundus shows an eccentrically located gestation sac (black arrow) on the left side of the uterine fundus, surrounded by a thin asymmetric myometrium separate from the endometrial cavity (white arrow) with no interstitial line sign

**Figure 2:**
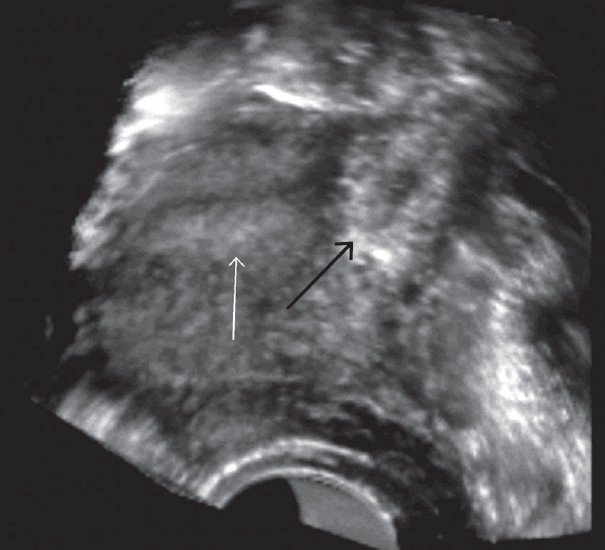
Coronal 3D scan shows a gestation sac (black arrow) in the left cornual region separate from the endometrial cavity (white arrow)

A 37 year-old para-2 woman with presenting complaints of irregular bleeding over 2–3 weeks following a menstrual delay of one week, was referred to our department for transvaginal ultrasonography. She had a history of prolonged cycles in the past but none of any previous ectopic pregnancy, pelvic inflammatory disease or the utilization of assisted reproductive techniques. Ultrasound examination revealed an empty uterine cavity with the evidence of a 4–5 week-old gestation sac (GS) located eccentrically on the left side of the uterine fundus [Figure 1]. The gestation sac revealed thin stripes of myometrial tissue present superiorly and inferiorly but not laterally. This raised the possibility of an interstitial ectopic pregnancy or an extrauterine tubal ectopic pregnancy adjacent to the uterus. Three-dimensional coronal ultrasound scans revealed the presence of the gestation sac in the fundal region located eccentrically on the left side in the region of cornu. This was suggestive of a high possibility of ectopic pregnancy located in the interstitial portion of the fallopian tube [Figure 2]. A urine pregnancy test performed after the ultrasound examination was found to be positive. Our patient was managed by conservative laparoscopic surgery, where the ectopic gestation sac located in the interstitial portion of the intact left fallopian tube was scooped out through an incision in the myometrium without resecting a portion of the tube. A local injection of vasopressin was used to control bleeding during excision of the ectopic gestation sac during laparoscopy. Methotrexate therapy was not considered as a treatment option in our case because of our center's lack of experience with the same and the high rate of success achieved with laparoscopy.

## DISCUSSION

Risk factors associated with the higher incidence of interstitial ectopic pregnancy include uterine anomalies, pervious ectopic pregnancy or salpingectomy, pelvic inflammatory disease, *in vitro* fertilization and ovulation induction. However, in our index case, none of the above risk factors were noted suggesting the role of natural causes as well.

Interstitial ectopic pregnancy is associated with a higher risk of shock and hemoperitoneum than other forms of ectopic pregnancy, as well as with a higher risk of maternal mortality due to delayed diagnosis and high vascularity of the myometrium.[2] The presence of an eccentrically located gestation sac with incomplete or asymmetric myometrial tissue, < 5 mm in thickness, is a highly suggestive but nonspecific indicator of interstitial pregnancy.[1] The presence of an echogenic line between the gestation sac and the endometrial cavity, also known as the interstitial line sign, is highly sensitive and specific for interstitial ectopic pregnancy.[3] Few reports exist of the utility of 3D transvaginal or endovaginal ultrasound (TVS or EVUS) in the diagnosis of interstitial ectopic pregnancy.[4] The 3D scans are very useful in obtaining the coronal scans of the fundal region of the uterus, giving a better overview of the cornual regions of the uterus. The characteristic features of an interstitial ectopic pregnancy (also seen in our case) include a GS located eccentrically outside the endometrial cavity of the uterus, in the region of the fundus with no or minimal identifiable myometrial tissue on its lateral aspect. This eccentric location and superior and lateral myometrial stripes are better and easily visualized on coronal scans generated through 3D TVS, an infrequent achievement with 2D scans.

Treatment options for interstitial ectopic pregnancy include local injection or systemic therapy with methotrexate, local injection of potassium chloride, conservative laparoscopic surgery and uterine artery embolism and in emergency situations, cornuectomy or hysterectomy.[5,6] Evidence of a hemorrhagic ectopic pregnancy is an indication for laparotomy.

To conclude, the diagnosis of an interstitial ectopic pregnancy is usually difficult and delayed resulting in high morbidity and mortality. In the present case, the diagnosis of an interstitial ectopic pregnancy was suggested on the basis of 3D transvaginal ultrasonography prior to the development of any complication, resulting in early management with a favorable outcome. This case thus highlights the role of 3D ultrasonography in cases of irregular bleeding or suspected early pregnancy, to diagnose or rule out unusual sites of ectopic pregnancy for their early safe management.
